# Employment of Small-Group Discussions to Ensure the Effective Delivery of Medical Education

**DOI:** 10.7759/cureus.52655

**Published:** 2024-01-21

**Authors:** Ankit Badge, Manju Chandankhede, Ujwal Gajbe, Nandkishor J Bankar, Gulshan R Bandre

**Affiliations:** 1 Microbiology, Datta Meghe Medical College, Datta Meghe Institute of Higher Education and Research, Nagpur, IND; 2 Biochemistry, Datta Meghe Medical College, Datta Meghe Institute of Higher Education and Research, Nagpur, IND; 3 Anatomy, Datta Meghe Medical College, Datta Meghe Institute of Higher Education and Research, Nagpur, IND; 4 Microbiology, Jawaharlal Nehru Medical College, Datta Meghe Institute of Higher Education and Research, Wardha, IND

**Keywords:** assessment strategies, facilitation techniques, critical thinking, active learning methods, medical education, small group discussion

## Abstract

The changing landscape of medical education has made small-group discussions crucial components. These sessions, including problem-based learning (PBL), case-based learning (CBL), and team-based learning (TBL), revolutionize learning by fostering active participation, critical thinking, and practical skills application. They bridge theory with practice, preparing future healthcare professionals for the dynamic challenges of modern healthcare. Despite their transformative potential, there are challenges in faculty preparation, resource allocation, and effective evaluation. The best practices include aligning discussions with curriculum goals, skilled facilitation, promoting active participation, and robust assessment strategies. Looking ahead, adapting to emerging health trends, ongoing research, and evolving healthcare demands will ensure the continued relevance and effectiveness of small-group discussions, shaping competent and adaptable healthcare providers equipped for the ever-evolving healthcare landscape.

## Introduction and background

Medical education is at the crossroads of tradition and innovation and addresses the changing needs of the 21st century [1]. In this rapidly changing landscape, the role of small-group discussions in medical education has become a powerful force of transformation, changing the way aspirant health professionals learn, collaborate, and adapt to modern medical demands [2]. These small interactive sessions, known as problem-based learning (PBL), case-based learning (CBL), or team-based learning (TBL), are becoming an essential component of medical curricula around the world, opening a new era of excellence in teaching [3]. The traditional model of medical education, characterized by didactic courses, repeated memory, and passive learning, has opened the way for a dynamic and participatory approach [4]. Medical educators recognize the need to encourage skills beyond the memory of facts; they seek to develop critical thinking, effective communication, and the application of knowledge in complex clinical scenarios [5]. Small-group discussions are an antidote to passive learning and provide a platform on which students can actively interact with medical concepts, exchange ideas, and deal with real patient cases [6]. The transformation of medical education is underway into an experience-based problem-solving effort, and small-group discussions are at the forefront of the process [7]. This review aims to highlight small-group discussions as a pedagogical tool to ensure the efficacy and depth of medical education.

## Review

Small-group discussions

Small-group discussions represent a pedagogical approach in which three to five students gather to participate in interactive, collaborative, and learning-centered activities [8]. Unlike traditional passive one-sided teaching, small-group discussions create an environment in which students actively participate in the learning process [9]. These meetings are characterized by open dialogue, critical thinking, problem-solving, and application of knowledge in real-world scenarios. They are versatile and adapt to various educational environments and subjects, which makes them especially suitable for medical education [10]. PBL, CBL, and TBL are structured forms of small group learning that take place in different forms in medical education, each offering unique advantages and customized learning approaches [11].

PBL is an approach that places students at the center of their education. In PBL, students are presented with a complex real-world problem or scenario associated with a medical case [12]. They collaborate to identify learning goals, generate hypotheses, conduct research, and ultimately solve problems. This method promotes critical thinking, self-directed learning, and problem-solving skills. It was developed in the 1960s at the McMaster University Medical School in Canada and has since gained international recognition and adoption [13,14]. CBL uses clinical cases and scenarios as a basis for discussions in small groups. Students analyze patient cases, discuss diagnostic and treatment strategies, and explore the underlying scientific principles. CBL is the bridge between theoretical knowledge and its practical application, allowing students to integrate medical understanding into real clinical situations [15]. TBL is a collaborative approach in which students work in teams to solve problems or discuss problems [16]. It usually involves a readiness assessment in which students individually prepare for a session, pass a readiness assessment test, and then participate in discussions and activities in a team. TBL promotes teamwork, critical thinking, and peer learning, essential skills in the medical profession [17].

Transition to active learning

The use of small groups in medical education dates back to the mid-20th century but was significantly recognized in the late 20th century and continued to develop. Traditionally, medical education relied heavily on didactic lectures. However, research in education and cognitive psychology shows that active learning methods, such as small-group discussions, are more effective in promoting deeper understanding and knowledge maintenance [18].

Medical students must not only acquire a deeper understanding of medical science but also learn how to apply this knowledge in clinical environments. Small-group discussions, particularly CBL and PBL, provide a bridge between theory and practice by offering students the opportunity to work in clinical situations and develop practical skills [19]. Small-group discussions are designed to promote critical thinking, clinical reasoning, and evidence-based decision-making skills. These are crucial skills for healthcare professionals who need to make complex, often life-changing decisions in their daily practice [20]. The modern medical landscape requires continuous learners who can adapt to the evolution of medical knowledge and technologies. Small-group discussions promote self-learning by encouraging students to take responsibility for their education and explore in-depth topics [21].

Small-group discussions with their different forms and historical developments are a pedagogical revolution in medical education [9]. These approaches provide students with a platform to actively engage in the material, develop essential skills, and prepare for the complex needs of contemporary health care. Their roots in active learning, clinical relevance, critical thinking, and self-directed learning are the cornerstones of modern medical education [22].

Benefits of small-group discussions

Small-group discussions play a key role in the development of critical thinking and clinical reasoning skills that are essential for physicians. By presenting complex clinical cases and problems to students, these sessions require them to analyze, synthesize, and apply their knowledge to practical situations [23]. Students are encouraged to think beyond memory and focus on understanding the underlying principles and their connection to patient care. This approach helps students develop the ability to effectively diagnose and treat patients, making them more competent and confident providers of healthcare [24].

Small-group discussions provide an ideal platform to promote evidence-based practice in medicine. Students are encouraged to critically evaluate the medical literature, research results, and clinical guidelines and then apply this knowledge to the cases or problems discussed [25]. They learn to recognize the quality of evidence, make informed decisions, and continuously update their practices based on recent research. This emphasis on evidence-based practice not only improves patient care but also promotes a culture of continuous learning and improvement among healthcare professionals [26]. The benefits of small-group discussions are tabulated in Table 1.

**Table 1:** Benefits of small-group discussions

Benefits of small-group discussions	Description
Improved ability to diagnose and treat patients	Enhances students' competence and confidence in diagnosing and treating patients by refining their practical problem-solving skills [24].
Promotion of evidence-based practice	Encourages the critical evaluation of medical literature, research findings, and clinical guidelines, promoting the reliance on empirical evidence in healthcare [25].
Critical evaluation of medical literature and research results	Encourages students to assess the quality and reliability of medical information, strengthening their ability to make evidence-based decision-making [25]
Application of knowledge to cases/problems discussed	Promotes the practical application of acquired knowledge to specific cases or problems addressed during discussions, reinforcing learning [25]
Recognition of evidence quality and informed decision-making	Develops the ability to discern evidence quality, enabling informed and evidence-driven decision-making in clinical practice [25]
Encouragement of continuous learning and improvement	Cultivates a culture of continuous learning, updating practices based on recent research, contributing to the professional growth of healthcare providers [26].
Enhancement of patient care	It leads to improved patient care outcomes by equipping healthcare professionals with enhanced skills and evidence-based approaches [26].

Side effects of small-group discussions

Time constraints represent a common concern, as discussions tend to extend beyond the allotted time, affecting other scheduled activities or agenda items. The presence of dominant participants poses another side effect, as specific individuals may assert control over the discussion, limiting the valuable contributions of others and potentially skewing perspectives. A lack of participation is possible, with participants exhibiting a hesitancy to express their opinions, thus impeding the overall richness of the discourse [27]. Differing perspectives within the group can lead to disagreements, which requires skillful facilitation skills for effective resolution. Additionally, the sharing of sensitive information raises confidentiality concerns, particularly pertinent in healthcare settings. Off-topic discussions may also emerge, diverting the conversation from its intended focus and potentially diminishing its effectiveness [7].

Improved communication skills

One of the fundamental skills of healthcare is the ability to communicate effectively with patients. Small-group discussions provide a safe and supportive environment for students to practice and improve their communication skills [28]. By discussing patient cases and scenarios, students learn how to communicate complex medical information clearly and compassionately, actively listen to patients' concerns, and establish trust and relationships. These skills are crucial to ensure that patients fully understand their conditions and treatment options and provide patient-centered care [29]. In modern healthcare, collaboration among teams and different health disciplines is essential to provide comprehensive and holistic care to patients. Small-group discussions often bring students from different fields of healthcare, such as medicine, nursing, pharmacy, and related health professions [30]. This interdisciplinary approach allows students to develop a greater appreciation for the roles and expertise of their colleagues. They learn how to collaborate effectively, share responsibility, and make team decisions. This not only prepares them for real interprofessional collaboration but also promotes a sense of unity and mutual respect between health professionals, ultimately in favor of patient results [31].

Small-group discussions focusing on critical thinking and communication serve as a bridge between theoretical knowledge and clinical practice. They empower students to think critically, analyze evidence, communicate effectively with patients, and communicate seamlessly with interprofessional teams. These skills are not only crucial to success in the medical field but also contribute to the overall quality of the medical service provided [32].

Potential challenges

A number of challenges have been identified in the smooth and effective conduct of small-group discussion sessions in the domain of medical education. One of the main challenges in implementing effective small-group discussions in medical education is the need for well-prepared faculty. Facilitators must have the skills necessary to guide discussions, encourage critical thinking, and provide constructive feedback. Faculty development programs are essential to ensure that teachers use active learning methods [33]. Training should include understanding the principles of adult learning, effective facilitation techniques, and the ability to adapt to different learning styles and group dynamics. Faculty members also need continuous support and resources to keep up with best practices and emerging educational technologies [34]. Small-group discussions may require a lot of resources and require appropriate places, materials, and technology to effectively facilitate the sessions. Institutions must allocate sufficient resources to create a conducive learning environment, provide a space equipped with technology, and provide access to relevant educational resources and materials [35]. This is particularly important in ensuring that the infrastructure meets the technological requirements of modern medical education, such as access to electronic health records and simulation tools. Resource constraints can prevent the implementation and success of small-group discussions in medical education [36]. Challenges in small-group discussions in medical education are presented in Table 2.

**Table 2:** Challenges in small-group discussions

Challenge	Description	Solutions
Faculty preparedness	The need for well-prepared faculty capable of leading discussions, fostering critical thinking, and providing constructive feedback [33].	Faculty development programs are crucial to imparting skills in active learning methods, understanding adult learning principles, effective facilitation, and adapting to diverse learning styles and group dynamics [34].
Resource allocation	Requires substantial resources for appropriate spaces, technology, and materials to create a conducive learning environment [35].	Institutions must allocate sufficient resources to ensure access to relevant educational materials, technological infrastructure, and equipped spaces for discussions [35].
Technology integration	The technological requirements of modern medical education are challenging, such as integrating electronic health records and simulation tools [36].	Resource limitations can hinder access to the necessary technology, limiting the successful implementation and efficacy of small-group discussions [36].

Student engagement and group dynamics

Medical students have different learning styles, preferences, and previous educational experiences. Some students can succeed in small groups, while others struggle to participate actively. Educators must take these differences into account and adopt strategies that adapt to different learning styles. This may include providing a mix of activities within small-group discussions, using multimedia resources, and providing flexible learning options to meet the needs of all students [37]. The dynamics of small groups can lead to conflict, imbalances in participation, or a lack of synergy between students. Some students may dominate discussions, while others may remain passive observers. Effective facilitation is essential to maintain a productive and respectful learning environment. The faculty must be qualified in the management of group dynamics, encourage the participation of all members, and solve conflicts constructively. Group dynamic techniques in education transform classrooms into experiential learning environments. Concepts related to human behavior are actively experienced through interactive processes, enabling health educators to foster understanding and appreciation of diverse values between school groups [38]. Techniques such as group contracts, peer assessments, and clear rules can help maintain a harmonious and equitable learning environment [39].

Assessment

Effective assessment strategies should include both formative and summative assessments. Formative assessments provide feedback during the learning process, allowing students to monitor progress and make the necessary adjustments. On the other hand, summative assessments evaluate overall outcomes and the effectiveness of small-group discussions in achieving educational objectives [40]. The development of reliable and valid assessment methods consistent with learning objectives and curriculum can be challenging but essential to ensure the quality of education of small-group discussions [41]. Formal assessments may include quizzes, group presentations, or reflection exercises, while summary assessments may include written assessments and performance assessments. A balanced combination of the two types of assessment ensures that students receive feedback on improvement and that overall educational objectives are met [42].

Fair and reliable assessment methods are essential to make a valid judgment on students' performances in small-group discussions. Assessments should take into account not only the individual contributions of students but also the effectiveness of the entire group. To ensure fairness and reliability, clear assessment criteria must be established, and teachers and students must be trained in the assessment process. In addition, the implementation of methods to reduce prejudices and maintain consistency in the classification is crucial [43]. Performance assessments can be an essential element of the assessment process. They encourage students to reflect on the contributions of their own peers, promote accountability, and promote self-assessment skills [44]. To overcome the challenges associated with small-group discussions in medical education, institutions, teachers, and students must exert a concerted effort. Faculty development, resource allocation, adaptation to different learning styles, group dynamics, and the establishment of effective assessment strategies are crucial components to ensure the success and sustainability of small-group discussions as the basic education methodology in medical programs [45].

Best practices in small-group discussions

Harmonization With Curriculum Objectives in Cognitive Skills Development

To ensure that discussions in small groups contribute effectively to general educational objectives, it is necessary to align these discussions with the objectives of the medical program curriculum. Learning objectives should be consistent with the larger objectives of the curriculum, ensuring that the content and skills discussed in small-group discussions are relevant and meet the educational outcomes of the program [46].

Effective Facilitation

Roles and abilities of facilitators: Effective facilitation is the cornerstone of a successful discussion in small groups. Faculty members who serve as facilitators must be well-versed in their roles, including guiding discussions, fostering a conducive learning environment, and providing timely feedback. The facilitator must have strong interpersonal skills, active listening, and the ability to adapt their approach to different groups and topics. In addition, they must be qualified to manage group dynamics and promote critical thinking among students [47].

Encouraging Active Participation

Facilitators should encourage and support the active participation of all students. This may involve employing various techniques, such as asking open questions, promoting discussions rather than lectures, and creating a safe space where students feel comfortable sharing their ideas. Active participation of students fosters critical thinking and improves the overall quality of the learning experience [48].

Designing Patient Cases or Scenarios

The design of a patient case or scenario is a key factor in small-group discussions, particularly PBL and CBL. Cases should be carefully constructed to challenge students to solve clinical problems in the real world while remaining educational and feasible. These cases serve as the basis for discussion and guide students through the diagnosis, treatment, and decision-making of evidence [49].

Creating a Meaningful Clinical Context

Small-group discussions should incorporate cases into a meaningful clinical context. In this context, students can link theoretical knowledge with practical applications and promote a deeper understanding of medical concepts. Realistic and authentic clinical scenarios also make learning experiences more interactive and relevant, thus improving student motivation and retention of information [24].

Group Dynamics and Teamwork

Small-group discussions inherently involve collaboration between students. Facilitators should promote the spirit of collaboration to ensure successful teamwork and emphasize the value of diverse perspectives and contributions. Implementing group agreements or guidelines for respectful communication can facilitate a productive and harmonious learning environment [50]. Group dynamics can sometimes lead to conflicts or challenges. Facilitators play a key role in constructively addressing these issues. Using conflict resolution strategies, encouraging open communication, and negotiating differences, facilitators can help students overcome obstacles and maintain a positive and collaborative environment in small groups [51].

Future directions

Adaptation to Emerging Health Trends

The health landscape is in constant evolution, influenced by advances in medical technology, changes in health policy, and changes in the population and needs. As small-group discussions are increasingly an integral part of medical education, teachers must adapt to these emerging health trends [52]. This adaptation involves reassessing the content of the curriculum to take into account the latest advances in medical research, such as precision medicine, telemedicine, artificial intelligence, and genomics. By incorporating these leading topics into small-group discussions, educators can ensure that students are prepared not only to face the challenges of modern medicine but also to encourage innovation and positive change in this area [53].

Research and Evidence-Based Approaches

The role of small-group discussions in medical education must be further refined and confirmed through ongoing research and evidence-based approaches. As educators and institutions adopt active learning methods, it is necessary to systematically assess the impact of these methods on student learning results, clinical competence, and patient care [54]. Research should investigate the most effective methods for implementing and evaluating small-group discussions, discover strategies to improve student participation and knowledge retention, and identify best practices for facilitation and evaluation. Evidence-based findings should guide the refinement of curriculum and teaching techniques to ensure that medical education remains at the forefront of educational research [55].

The Continuing Evolution of Medical Education

The evolution of medical education goes far beyond the inclusion of discussion in small groups. This field is undergoing a transformation process driven by the recognition that health professionals need to have a broader set of skills, including interprofessional collaboration, cultural competence, and adaptability [56]. Small-group discussions play an important role in preparing students for these evolving requirements by encouraging communication skills, teamwork, and adaptability. As medical education continues to evolve, institutions can explore innovative approaches such as hybrid learning models, changing classrooms, and competency-based education to complement traditional methods and meet student needs [57].

The future of small-group discussions in medical education is promising, as long as they remain flexible, adaptable, and responsive to the dynamic nature of health care. Through an understanding of emerging health trends, investing in research and evidence-based practices, and supporting the ongoing evolution of medical education, educators can ensure that small-group discussions remain a cornerstone of the medical curriculum, and promote competent, compassionate, and adaptable healthcare professionals [58]. These discussions will continue to shape the future of physicians and healthcare providers, equipping them to meet the complex needs of patients and the ever-changing healthcare landscape [59].

## Conclusions

Small-group discussions are not just a pedagogical tool but a dynamic force for change in medical education. They are essential for cultivating critical thinkers, effective communicators, and collaborative practitioners that healthcare demands. By embracing their crucial role and remaining committed to ongoing research and improvement, educators can harness the full potential of small-group discussions, producing graduates who are knowledgeable and ready to make a real difference in the lives of patients and the field of medicine itself. Through small-group discussions, students can actively engage with the material and learn from the perspectives of their peers. This interactive approach fosters a deeper understanding of complex medical concepts and encourages students to think critically about different treatment approaches. Small-group discussions provide a safe space for students to practice communication skills and develop empathy, which is crucial to building effective doctor-patient relationships. By incorporating ongoing research and student feedback, educators can continually improve the effectiveness of small-group discussions and ensure that medical education is in line with the evolving needs of the healthcare industry.
